# Effects of winter irrigation on soil salinity and jujube growth in arid regions

**DOI:** 10.1371/journal.pone.0218622

**Published:** 2019-06-26

**Authors:** Zhipeng Liu, Xiyun Jiao, Shenghan Lu, Chengli Zhu, Yaming Zhai, Weihua Guo

**Affiliations:** 1 College of Agricultural Engineering, Hohai University, Nanjing, Jiangsu, P.R. China; 2 State Key Laboratory of Hydrology-Water Resources and Hydraulic Engineering, Hohai University, Nanjing, Jiangsu, P.R. China; Harran University, Sanliurfa, TURKEY

## Abstract

The considerably high evapotranspiration and the low leaching fraction of the soil in arid regions are likely the primary causes of the enhanced soil salinity in such regions. Winter irrigation has proven to be very effective for promoting the leaching of salts from the rooting-zone. In this study, we investigated the effects of different irrigation methods (flood irrigation and drip irrigation) and winter irrigation quotas (450, 1350, 2250, 3150, 4050, and 4950 m^3^/hm^2^) on soil salinity and plant growth in an arid region. The sum of *EC*_e_ in the 0–100 cm soil layer was 56.26–29.32 ms/cm under flood irrigation, 61.37–17.90 ms/cm under drip irrigation, and 64.13 ms/cm under no irrigation. The survival rates of jujube trees reached 65% and 77%, respectively, for drip irrigation and flood irrigation with a quota of 2250 m^3^/hm^2^. Furthermore, at irrigation quotas in excess of 3150 m^3^/hm^2^ the ground diameter and height of jujube trees were significantly greater than those observed under nonwinter irrigation and several other winter irrigation treatments. These findings indicated that winter irrigation significantly reduced soil salinity, changed the soil salt distribution, created a good environment for the growth of jujube trees and improved the survival rate of young jujube trees, especially under winter drip irrigation with a quota of 3150 m^3^/hm^2^. In addition, 1-year-old jujube trees emerging in spring may benefit from an *EC*_e_ lower than 5 ms/cm.

## Introduction

Arid regions receive minor amounts of rainfall and experience high atmospheric demand for water vapor [1]. Unsurprisingly, irrigation plays a significant role in agricultural production in arid regions [2,3]. In general, agricultural irrigation accounts for more than 90% of the total water consumption [4,5] globally, and these trends are expected to remain unchanged in the foreseeable future. Over the past decades, it has been recognized that sustained irrigation may brings about land salinization, especially in arid regions [6–8]. The results of a 2-year experiment by Liu et al. [9] showed that salts mainly accumulated in the 0~60 cm soil layer during a growing season.

Soil salinization is a main cause of poor ecological environments and prevents the development of sustainable agriculture [7,10,11]. Leaching of soil salt during the nongrowth period has been proposed as a possible mitigation practice and has received renewed research interest [9,12–14]. The premise of this proposal is that winter irrigation will be conducted to elicit leaching of salts from the rooting-zone. Because the atmospheric demand for water vapor is low and evaporative losses are small, winter irrigation has been recommended for moderate or severe salinization to reduce farmland salinity [9,15–17]. Chen et al. [12] used a revised ENVIRO-GRO model to show that flood irrigation with a large amount of water after harvest can efficiently and significantly reduce salt accumulation within the top soil profile. Similarly, Yang et al. [18] reported that winter irrigation is one of the water and salt management practices widely adopted in arid irrigated areas and that the strength of the desalination effect of winter irrigation increased with increasing water irrigation volume. Zhong et al. [16] recommended that winter irrigation should be carried out when the ground begins to freeze at night but thaws during the day and reported that the beneficial effects of winter irrigation arise mainly from the washing away of salts, which decreases salinity. In addition, Liu et al. [9] found that after a 150-mm winter irrigation application, the salts that had accumulated in the 0~60 cm layer were leached into the deeper soil layers and that the soil salt content as measured by its electrical conductivity (*EC*_1:5_) decreased to approximately 0.2 dS/m in the following year. Therefore, the amount of water leached during the nongrowth period that can effectively prevent secondary salinization of soil and improve sustainable use of land should be taken into account when designing winter irrigation practices [19–21].

Although some positive effects of winter irrigation have been confirmed by previous experimental studies and model simulations, most researchers have focused on how winter irrigation affects the moisture and salt content of the soil. Thus, how winter irrigation affects soil salinity, survival rate and growth index in the following year remains a subject of scientific inquiry. The objectives of this study are (1) to investigate the effects of different irrigation methods and winter irrigation quotas on soil salinity and plant growth; and (2) to obtain information that can inform the development of for irrigation management practices aiming to prevent secondary soil salinization in arid regions.

## Materials and methods

### Experimental site

A field experiment was conducted continuously from early November 2017 to late July 2018. The experimental site is situated in Qiemo County, Bayingolin, Xinjiang Province, Northwest China (latitude: 37°47' N, longitude: 84°08' E, altitude: 1307 m), which has a continental arid climate with a mean annual precipitation of 18.1 mm and a mean annual evaporation of 2824 mm. The soil is classified as sand (USDA) and consists of 86.48% sand, 10.45% silt and 3.07% clay. The average soil bulk density from the surface to a depth of 1.0 m is approximately 1.47 g/cm^3^, with a field capacity of 15.13% (mass basis). The groundwater depth is 2.2 m, and the groundwater is of high salinity (3.17 g/L). The the irrigation water comes from the fresh (0.47 g/L) snowmelt water of Kunlun Mountain. The monthly precipitation, relative humidity and air temperature during the study period are provided in Table 1.

**Table 1:** Air temperature, relative humidity and precipitation data of the study site.

Month	Mean temperature (°C)	Maximum temperature (°C)	Minimum temperature (°C)	Relative humidity (%)	Precipitation (mm)
November	1.7	23.2	-15.2	35.5	0.0
December	-4.8	10.0	-18.1	57.6	1.9
January	-7.8	17.3	-21.2	47.0	0.0
February	-3.0	17.2	-20.0	43.0	0.0
March	11.3	29.7	-6.5	22.3	0.0

### Experimental design

**Fig 1:**
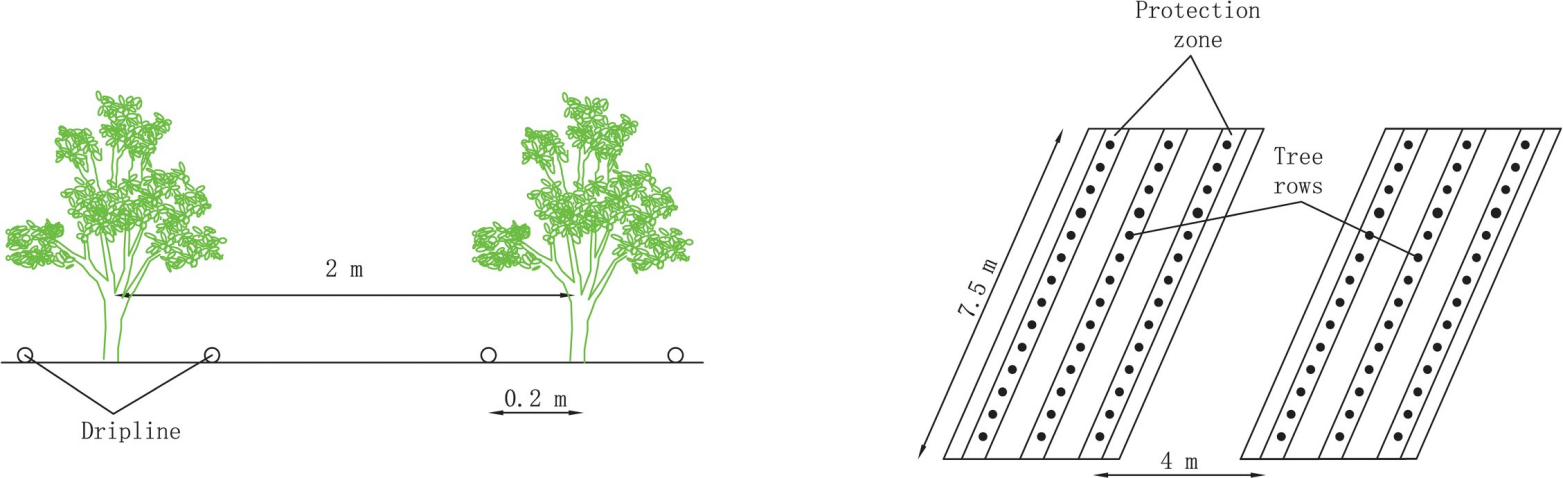
Layout of the drip irrigation design in this study.

This experiment used 1-year-old *Zizyphus jujube* trees that had been planted in rows 0.3 m apart with a spacing of 2 m within each row, and it involved a completely randomized design with three replicate plots. Each replicate plot was 15 m^2^ (2 m wide and 7.5 m long) and contained 25 trees arrayed in the same row (Fig 1). To avoid water and salt movement across plots, the distance between adjacent plots was 4 m. The primary laterals of 1-year-old *Zizyphus jujube* trees are less than 20 cm. Two driplines provided water without pressure compensation on both sides of each tree row and were located 0.2 m away from the row (with two emitters separated by a 0.3 m distance, 3.2 L/h of water provision per emitter, and a manufacturing coefficient of variation ≤5%). The treatments included drip irrigation (DI) and flood irrigation (FI) with 6 irrigation quotas (450, 1350, 2250, 3150, 4050, and 4950 m^3^/hm^2^). In addition, a treatment without winter irrigation was established for the control group (CK). In total, 13 treatments were designed and applied. In each plot, a flow meter was installed to control the irrigation amount. After winter irrigation, all treatment plots received identical applications of irrigation, fertilization, pruning, and insecticides.

### Soil salt content measurements

Soil samples were randomly collected at depths of 0–20 cm, 20–40 cm, 40–60 cm, 60–80 cm and 80–100 cm from the soil surface by a soil auger on 3 November (before winter irrigation), 11 November (7 days after winter irrigation) and 5 April (end of the rest period). The soil samples were air-dried, ground and sieved to determine the soil salt content. A digital conductivity instrument (FE30K Plus, Mettler Toledo Corporation, China) was used to measure the electrical conductivity of the saturated soil extract (*EC*_*e*_) [21].

### Growth measurements

The total number of plants and the number of surviving plants were calculated based on 20 trees in the middle of the row; this middle trees were selected to eliminate boundary effects. These numbers were calculated in May 2018 and used to calculate the survival rate (number of surviving plants/ total number of plants) [22]. Twenty tag-labeled jujube trees were selected from the three replicates of each treatment to measure and calculate the jujube tree growth from late April to late July. The height and ground diameter of jujube trees were measured by a steel ruler and Vernier caliper, respectively, twice per month. The average cumulative growth of each jujube tree was calculated based on the first and last measurements of height and ground diameter.

### Statistical analysis

Analysis of variance (ANOVA) and correlation analysis were performed using Statistical Analysis Software (SPSS 20.0, International Business Machines Corporation (IBM), USA), and the means of different treatments were tested for significant differences using Duncan’s multiple range test at a significance level of P_0.05_ and P_0.01_.

## Results

### Soil salinity

**Fig 2:**
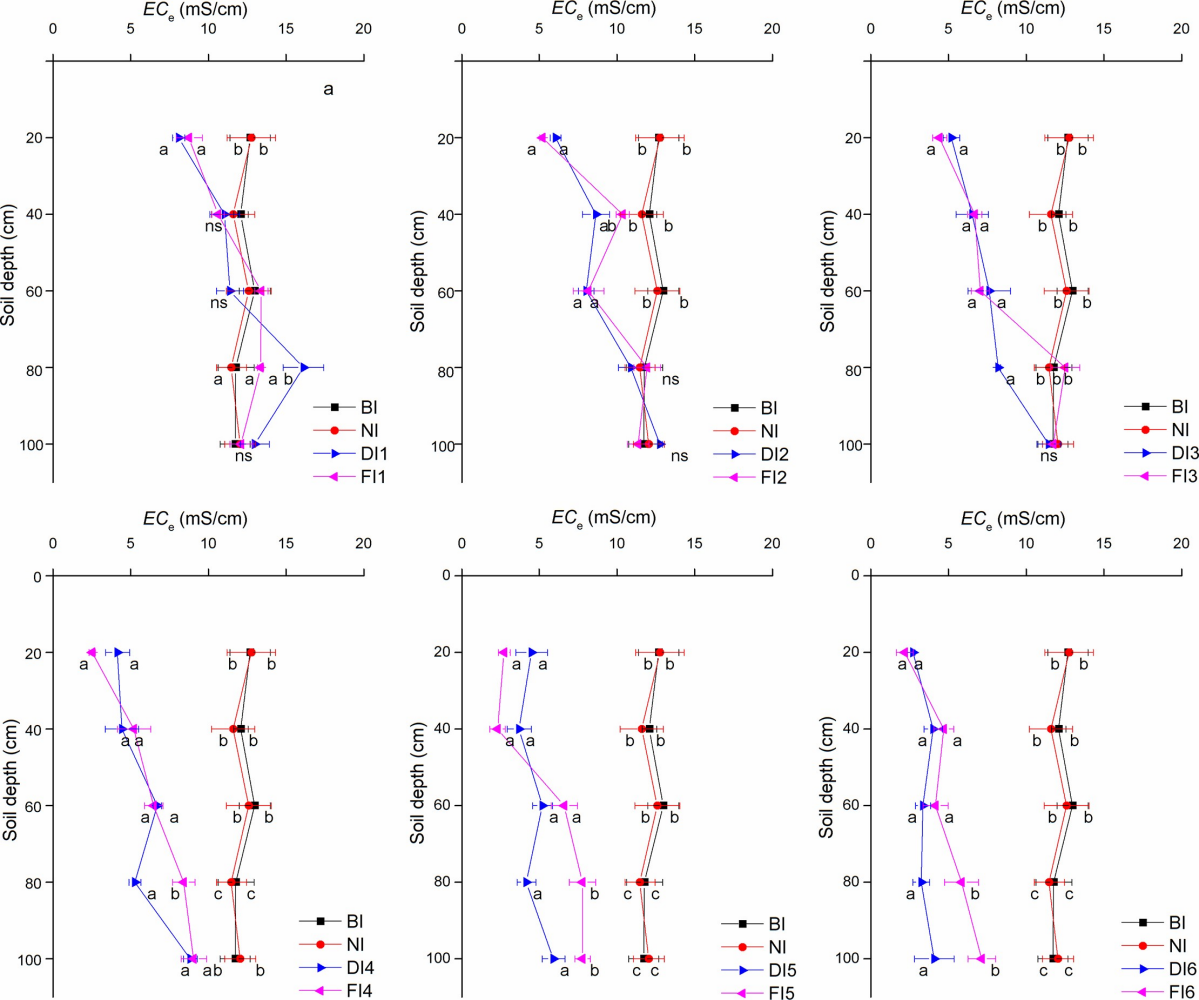
*EC*_e_ values seven days after winter irrigation. BI, before irrigation; NI, no irrigation. DI1-DI6 represent the drip irrigation treatments with irrigation quotas of 450, 1350, 2250, 3150, 4050 and 4950 m^3^/hm^2^. FI1-FI6 represent the flood irrigation treatments with irrigation quotas of 450, 1350, 2250, 3150, 4050 and 4950 m^3^/hm^2^. Values represent the mean ± SE of at least three replicates. Significant differences are indicated by different small letters among treatments at a given soil depth (P < 0.05), and ns represents no significant difference (P > 0.05).

The soil salinity measured seven days after winter irrigation was decreased relative to that measured concurrently in the control plots, especially in the surface soil (Fig 2). Compared with no irrigation (NI), *EC*_e_ in the 0–20 cm soil was 4.00, 7.55, 8.33, 10.23, 10.01 and 10.57 ms/cm lower under FI and 4.67, 6.70, 7.57, 8.60, 8.25 and 10.03 ms/cm lower under DI. However, *EC*_e_ of FI1 and DI1 in the 60–80 cm soil was 16.39% and 40.45% higher, respectively, than that in NI. In FI2 and DI2, *EC*_e_ in the 20–60 cm soil was 1.72–4.95 ms/cm lower than that before irrigation (BI). *EC*_e_ in the 20–80 cm soil was 6.68, 7.04, and 12.51 ms/cm in FI3 and 6.51, 7.62, and 8.20 ms/cm in DI3. In the 0–100 cm soil layer, *EC*_e_ of each of FI4, FI5, FI6 DI4, DI5 and DI6 was significantly lower than that under NI and BI. The decreases in *EC*_e_ increased with an increasing irrigation quota. Compared with the sum of *EC*_e_ in the 0–100 cm soil under BI, that in FI4, FI5 and FI6 was reduced by 48.26%, 55.53% and 60.81%, respectively, and that in DI4, DI5 and DI6 declined by 52.00%, 61.64% and 71.59%. It can be concluded that FI resulted in a higher *EC*_e_ than did DI under the same winter irrigation quota.

**Fig 3:**
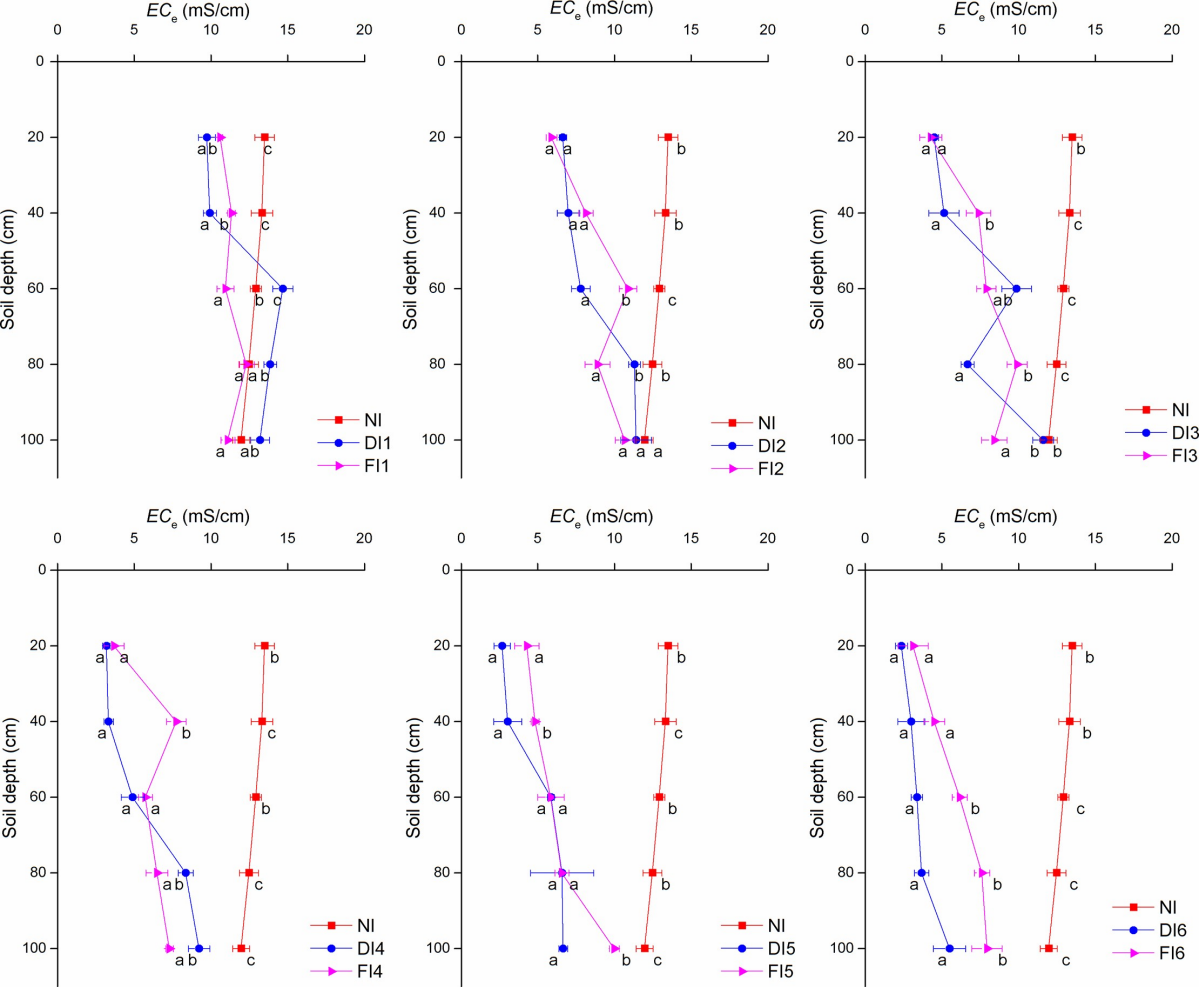
*EC*_e_ values before sprouting the following year. Values represent the mean ± SE of at least three replicates. Significant differences are indicated by different small letters among treatments at a given soil depth (P < 0.05), and ns represents no significant difference (P > 0.05).

The soil salinity before sprouting in the following year was similar to that seven days after winter irrigation (Fig 3). The sum of *EC*_e_ in the 0–100 cm soil was 56.26, 44.43, 37.83, 30.89, 31.48 and 29.32 ms/cm under FI and 61.36, 44.12, 37.76, 28.97, 24.80 and 17.89 ms/cm under DI. Under NI, the sum of *EC*_e_ was 64.13 ms/cm. The results showed that the soil salinity before sprouting in the following year was significantly affected by the different winter irrigation treatments.

### Survival rate

**Fig 4:**
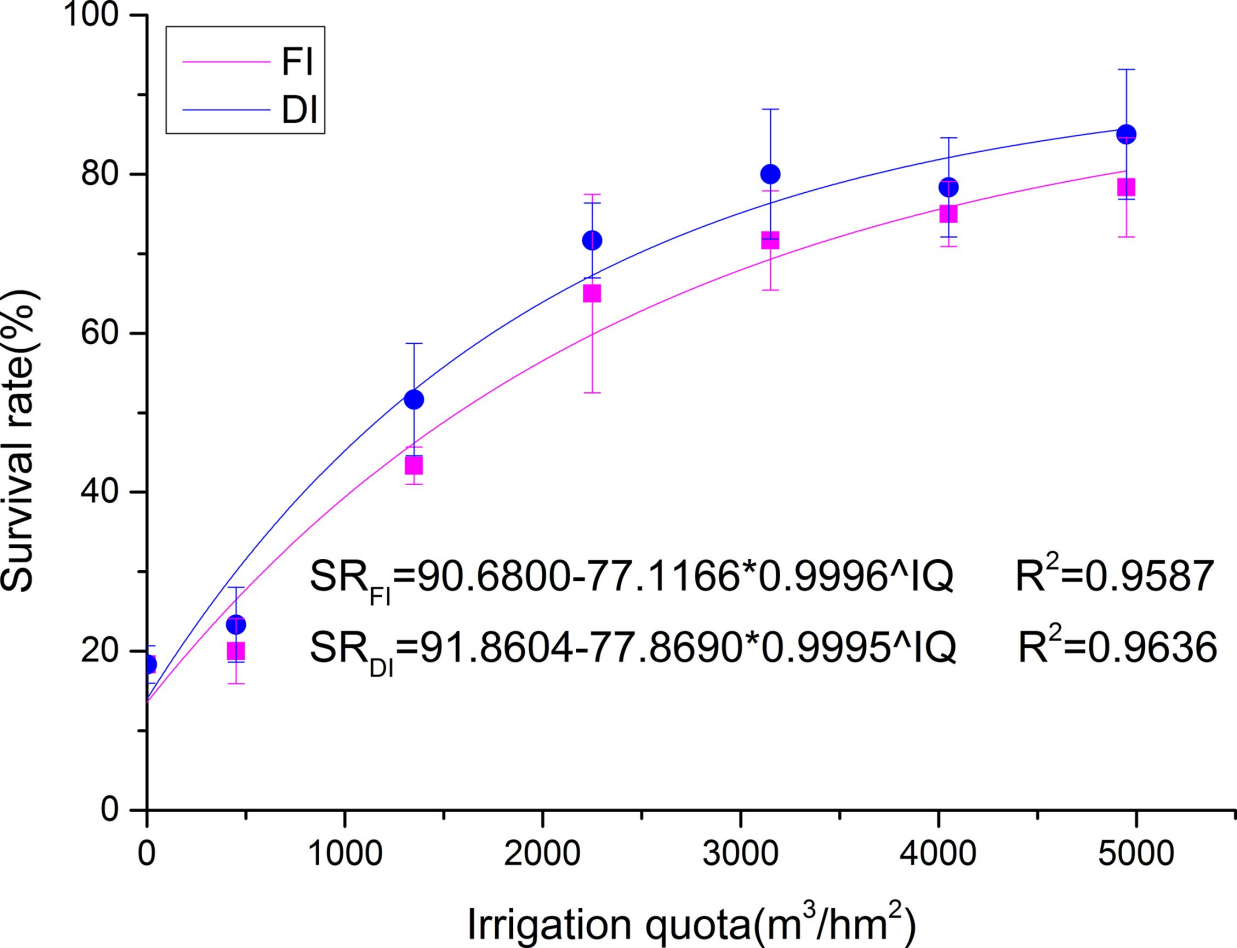
Survival rate in different treatments in the following year. The fitted curves for irrigation quota against the survival rate of jujube trees obtained from tests are plotted. Values represent the mean ± SE (n = 3).

With an increase in the irrigation quota, the survival rate of young jujube trees significantly improved in the following year (Fig 4). The survival rate of jujube trees was highest for those irrigated with 4950 m^3^/hm^2^, reaching approximately 80%. However, the survival rates in FI3 and DI3 reached 65% and 72%, respectively. This result indicated that the survival rate under DI was slightly higher than that under FI under the same irrigation quota.

### Ground diameter and height

**Fig 5:**
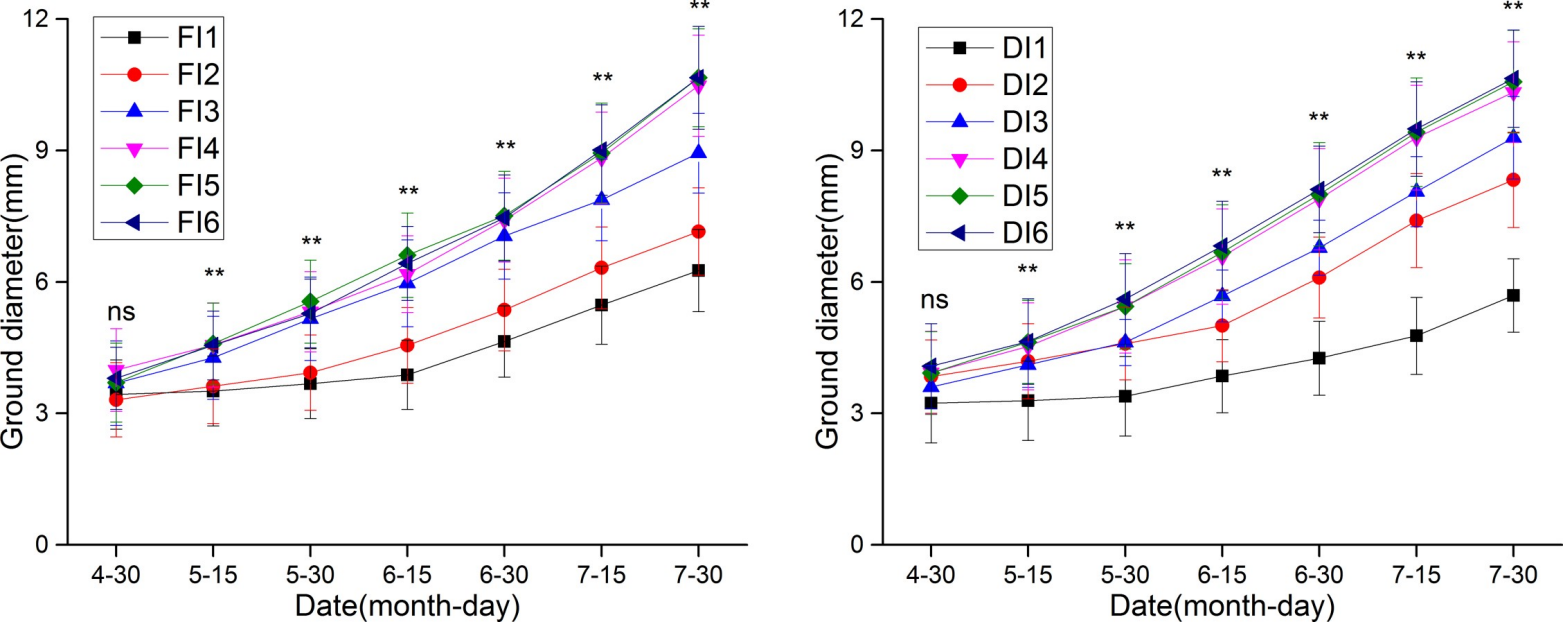
The ground diameter of jujube trees under different treatments. Values represent the mean ± SE (n = 20). Above the error bars, * denotes a significant difference (P < 0.05), ** denotes a very significant difference (P < 0.01), and ns denotes no significant difference (P > 0.05).

**Fig 6:**
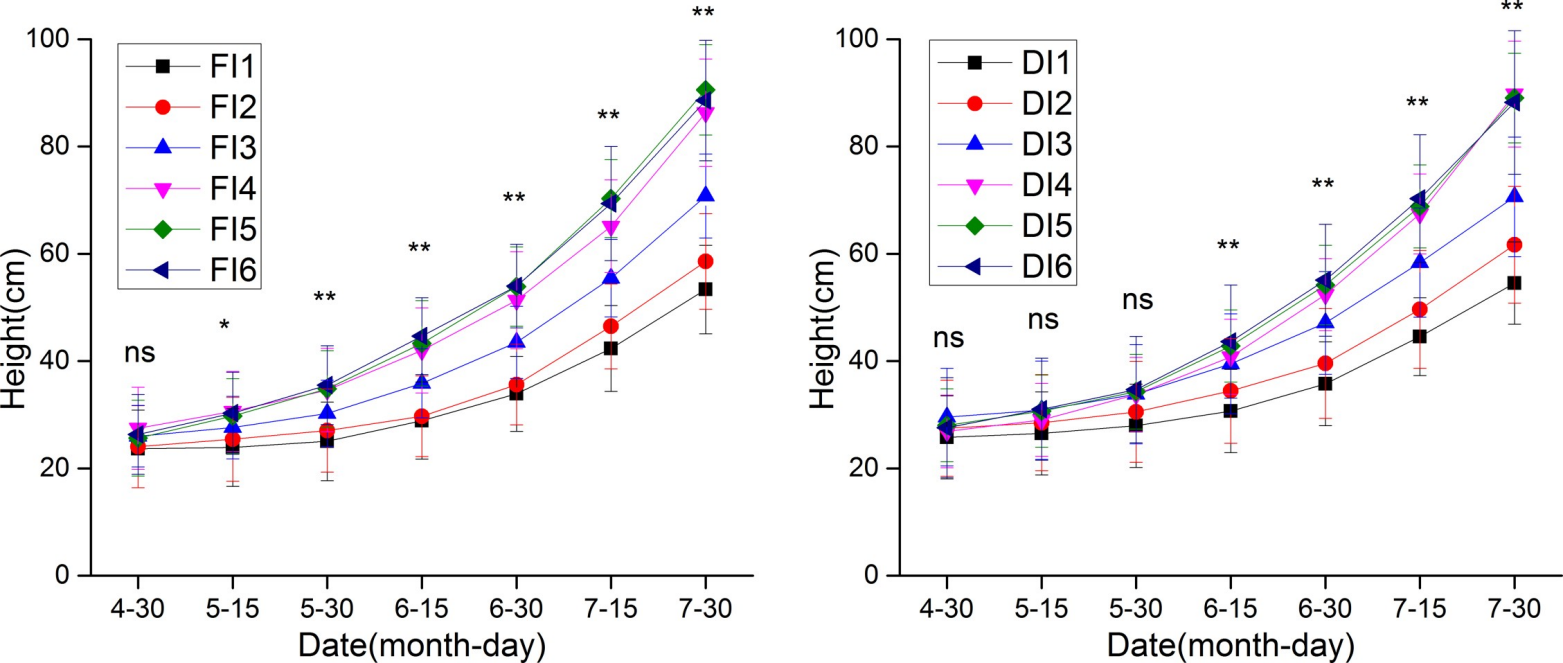
Height of jujube trees under different treatments. Values represent the mean ± SE (n = 20). Above the error bars, * denotes a significant difference (P < 0.05), ** denotes a very significant difference (P < 0.01), and ns denotes no significant difference (P > 0.05).

The ground diameter and height of jujube trees continuously increased from late April to late July (Figs 5 and 6). Comparisons revealed no significant differences among treatments in ground diameter or height in late April. However, in late July, the ground diameter in FI2-FI6 was 0.89, 2.68, 4.22, 4.40 and 4.40 mm higher than that in FI1, and that in DI2-DI6 was 2.64, 3.60, 4.64, 4.88 and 4.95 mm higher than that in DI1. By 30 July, the height in FI1-FI6 had increased to 53.35, 58.58, 70.80, 86.30, 90.57 and 88.57 cm, and that in DI1-DI6 had increased to 54.54, 61.67, 70.62, 89.79, 89.06 and 88.22 cm. The findings indicated that irrigation quota had a significant effect on the ground diameter and height of jujube trees.

## Discussion

### Soil salinity

Different irrigation methods in winter impact the distribution and content of soil salt [9,14,15]. In the low-irrigation-quota (450, 1350 and 2250 m^3^/hm^2^) treatments (Fig 2), the soil salinity in the upper layer decreased; however, soil salinity in the deep layer was less affected by irrigation, being not significantly different from or being higher than that in the treatment without winter irrigation. However, in the high-irrigation-quota (3150, 4050 and 4950 m^3^/hm^2^) treatments, the soil salinity at a depth of 0~100 cm was significantly lower than that in the treatment without winter irrigation. Similar results have been found in other studies [9,18,23]. However, the salt content of the soil under flood irrigation was higher than that under drip irrigation at a depth of 0~100 cm. This result agrees with that of Zhao et al. [24], who observed that the soil salinity under flood irrigation was higher than that under drip irrigation at a depth of 0~200 cm for a given irrigation quota. This result may be due to the fact that infiltration under drip irrigation is closer to uniform than that under flood irrigation and that soil salt constantly migrates to the deep layer with water movement.

The soil salt content in each treatment in the following spring is shown in Fig 3. The soil salinity remained significantly affected by winter irrigation before jujubes emerged in the spring. Zhao et al. [24] reported similar results in cotton. The soil salt distribution at a depth of 0~100 cm remained similar to that measured 7 days after winter irrigation. In contrast, Yang et al. [18] reported that the effects of winter irrigation on water storage and salinity control in all the studied treatments decreased gradually with time. A low air temperature and high relative humidity (Table 1) may result in low soil evaporation; this phenomenon was observed in our study. As reported by Shang et al. [25], only a small part of winter irrigation water is consumed by soil evaporation; most of it is stored in the soil, increasing the soil moisture content.

The results demonstrate that due to the salt-leaching effects and the water use efficiency of winter irrigation, proper irrigation quotas of winter irrigation effectively reduced the soil salinity. Excessive irrigation is not inappropriate, especially in arid regions. According to the content and distribution characteristics of soil salinity in Figs 2 and 3, the appropriate irrigation quota is 3150 m^3^/hm^2^. However, due to the freezing and thawing in the soil layer, soil microorganisms [26], plant diseases [27] and insect pests [28] are also affected. Therefore, winter irrigation not only influences the soil salinity in the following spring but also affects subsequent crop growth and development. Hence, it is necessary to analyze the influences of different irrigation methods and quotas on the growth and yield of crops (such as jujube, a typical crop in Xinjiang) in the following year.

### Survival rate

Plant seedlings are more severely affected by salinity stress than are older plants because seedling roots occur in the upper layer of soil [8]. For 1a jujube, survival rate is the most important indicator of whether an irrigation method is effective (Fig 4). The different irrigation quotas played important roles in improving the survival rate of jujube, whereas irrigation method had weaker effects. Our research confirmed that winter irrigation, especially drip irrigation with a large amount of water, created a good environment for the growth of jujube trees and improved the survival rate of young jujube trees. These results agree with those of Shao et al. [13], Liu et al. [9] and Chen et al. [12], who found that winter irrigation created a good environment in the upper soil for crop growth. Moreover, Zhao et al. [24] showed that winter irrigation can help maintain the soil moisture content and improve the rate of seedling emergence, especially irrigation with a relatively large amount of water. Thus, the higher survival rate of young jujube trees under adequate irrigation could be a consequence of the higher soil moisture content; a the low availability of moisture at the sowing stage adversely affects the germination, seedling establishment and growth of plants [29]. Moreover, the survival rate of jujube trees was higher under drip irrigation than under flood irrigation.

### Ground diameter and height

The ground diameter and height of jujube trees under winter irrigation were significantly higher than those of the control trees (Figs 5 and 6). Comparisons of jujube ground diameter and height among the irrigation treatments revealed that winter irrigation had important effects on crop growth and development in the following year. Under drip irrigation and flood irrigation, both the ground diameter and height of jujube were positively correlated with irrigation quota. However, when the irrigation quota exceeded 3150 m^3^/hm^2^, ground diameter and height were significantly higher than they were under nonwinter irrigation and several other winter irrigation treatments, indicating that the jujube trees under this quota underwent vigorous growth. These findings are consistent with the findings of Zhao et al. [29] who reported that winter drip irrigation with a quota of 3000 m^3^/hm^2^ improved the canopy photosynthetic potential, group net assimilation rate and leaf area index of cotton by 34.30%, 19.23% and 42.60%, respectively, relative to the corresponding values under treatment without winter irrigation. Furthermore, the ground diameter and height under DI were slightly higher than those under FI at the same irrigation quota. Therefore, an appropriate winter irrigation quota can effectively promote the growth and development of jujube in the following year, especially under drip irrigation.

## Conclusions

In summary, winter irrigation should be carried out to decrease soil salinity and promote crop growth and development under arid climatic conditions. Irrigation quota played an important role in influencing the soil salinity distribution, and the amplitude of the effect of irrigation was positively correlated with the irrigation quota.

Considering the influence of soil salinity and the observed survival rates and growth index values in the following year, the winter irrigation quota of approximately 3150 m^3^/hm^2^ is most appropriate under the conditions of this experiment. This study showed that 1a jujube trees are sensitive to salt in the soil, that different salt contents had different influences, and that *EC*_e_ should be lower than 5 ms/cm when jujube trees emerge in the spring. However, soil freezing and thawing in the winter is a complex natural phenomenon with effects on soil microorganisms, plant diseases and insect pests. Therefore, the effects of soil freezing and thawing on soil salinity and plant characteristics require more research in the future.

## References

[1] Gui DW, Lei JQ, Zeng FJ. Farmland management effects on the quality of surface soil during oasification in the southern rim of the Tarim Basin in Xinjiang, China. Plant, Soil and Environment. 2010; 56:348–356. 10.17221/54/2009-PSE

[2] Ma L, Liu X, Wang Y, Wu P. Effects of drip irrigation on deep root distribution, rooting depth, and soil water profile of jujube in a semiarid region. Plant and Soil. 2013; 373:995–1006. 10.1007/s11104-013-1880-0

[3] Wang Z, Wu Q, Fan B, Zheng X, Zhang J, Li W, et al Effects of mulching biodegradable films under drip irrigation on soil hydrothermal conditions and cotton (Gossypium hirsutum L.) yield. Agricultural Water Management. 2019; 213:477–485. 10.1016/j.agwat.2018.10.036

[4] Shen Y, Li S, Chen Y, Qi Y, Zhang S. Estimation of regional irrigation water requirement and water supply risk in the arid region of Northwestern China 1989–2010. Agricultural Water Management. 2013; 128:55–64. 10.1016/j.agwat.2013.06.014

[5] Huang Y, Li YP, Chen X, Ma YG. Optimization of the irrigation water resources for agricultural sustainability in Tarim River Basin, China. Agricultural Water Management. 2012; 107:74–85. 10.1016/j.agwat.2012.01.012

[6] Schilfgaarde J. Irrigation—a blessing or a curse. Agricultural Water Management. 1994; 25:203–219.

[7] Wang Y, Xiao D, Li Y, Li X. Soil salinity evolution and its relationship with dynamics of groundwater in the oasis of inland river basins: case study from the Fubei region of Xinjiang Province, China. Environmental Monitoring and Assessment. 2008; 140:291–302. 10.1007/s10661-007-9867-z 17690990

[8] Aslam M, Maqbool MA, Zaman QU, Shahid M, Arslan Akhtar M, Rana AS. Comparison of Different Tolerance Indices and PCA Biplot Analysis for Assessment of Salinity Tolerance in Lentil (Lens culinaris) Genotypes. International Journal of Agriculture and Biology. 2017; 19:470–478. 10.17957/IJAB/15.0308

[9] Liu M, Yang J, Li X, Liu G, Yu M, Wang J. Distribution and dynamics of soil water and salt under different drip irrigation regimes in northwest China. Irrigation Science. 2013; 31:675–688. 10.1007/s00271-012-0343-3

[10] Li X, Jin M, Zhou N, Huang J, Jiang S, Telesphore H. Evaluation of evapotranspiration and deep percolation under mulched drip irrigation in an oasis of Tarim basin, China. Journal of Hydrology. 2016; 538:677–688. 10.1016/j.jhydrol.2016.04.045

[11] Zhang Z, Hu H, Tian F, Hu H, Yao X, Zhong R. Soil salt distribution under mulched drip irrigation in an arid area of northwestern China. Journal of Arid Environments. 2014; 104:23–33. 10.1016/j.jaridenv.2014.01.012

[12] Chen W, Hou Z, Wu L, Liang Y, Wei C. Evaluating salinity distribution in soil irrigated with saline water in arid regions of northwest China. Agricultural Water Management. 2010; 97:2001–2008. 10.1016/j.agwat.2010.03.008

[13] Shao LW, Zhang XY, Sun HY, Chen SY, Wang YM. Yield and water use response of winter wheat to winter irrigation in the North China Plain. JOURNAL OF SOIL AND WATER CONSERVATION. 2011; 66:104–113. 10.2489/jswc.66.2.104

[14] Wang R, Kang Y, Wan S, Hu W, Liu S, Liu S. Salt distribution and the growth of cotton under different drip irrigation regimes in a saline area. Agricultural Water Management. 2011; 100:58–69. 10.1016/j.agwat.2011.08.005

[15] Bing H, He P, Zhang Y. Cyclic freeze–thaw as a mechanism for water and salt migration in soil. Environmental Earth Sciences. 2015; 74:675–681. 10.1007/s12665-015-4072-9

[16] Zhong R, Tian F, Yang P, Yi Q. Planting and Irrigation Methods for Cotton in Southern Xinjiang, China. Irrigation and Drainage. 2016; 65:461–468. 10.1002/ird.2015

[17] Forkutsa I, Sommer R, Shirokova YI, Lamers JPA, Kienzler K, Tischbein B, et al Modeling irrigated cotton with shallow groundwater in the Aral Sea Basin of Uzbekistan: II. Soil salinity dynamics. Irrigation Science. 2009; 27:319–330. 10.1007/s00271-009-0149-0

[18] Yang P, Zia-Khan S, Wei G, Zhong R, Aguila M. Winter Irrigation Effects in Cotton Fields in Arid Inland Irrigated Areas in the North of the Tarim Basin, China. Water. 2016; 8:47 10.3390/w8020047

[19] Hu H, Tian F, Zhang Z, Yang P, Ni G, Li B. Soil salt leaching in non—growth period and salinity dynamjcs under mulched drip irrigation in arid area. Journal of Hydraulic Engineering. 2015; 9:1037–1046.

[20] Forkutsa I, Sommer R, Shirokova YI, Lamers JPA, Kienzler K, Tischbein B, et al Modeling irrigated cotton with shallow groundwater in the Aral Sea Basin of Uzbekistan: I. Water dynamics. Irrigation Science. 2009; 27:331–346. 10.1007/s00271-009-0148-1

[21] Burt CM, Isbell B. Leaching of accumulated soil salinity under drip irrigation. TRANSACTIONS OF THE ASAE. 2005; 48:2115–2121.

[22] Zhang C, Li X, Kang Y, Wang X. Salt leaching and response of Dianthus chinensis L. to saline water drip-irrigation in two coastal saline soils. Agricultural Water Management. 2019; 218:8–16. 10.1016/j.agwat.2019.03.020

[23] Chen H, Peng Z, Zeng W, Wu J. Salt Movement during Soil Freezing Events in Inner Mongolia, China. Journal of Coastal Research. 2018; 82:55 10.2112/SI82-007.1

[24] Zhao B, Wang Z, Li W. Effects of winter drip irrigation mode and quota on water and salt distribution in cotton field soil and cotton growth next year in northern Xinjiang. Transactions of the Chinese Society of Agricultural Engineering. 2016; 6:139–148.

[25] Shang S, Lei Z, Yang S. Numerical Simulation on the Effect of Winter Irrigation on Soil Moisture Regime in Winter. Transactions of the Chinese Society of Agricultural Engineering. 1997; 3:70–75.

[26] Han C, Gu Y, Kong M, Hu L, Jia Y, Li F, et al Responses of soil microorganisms, carbon and nitrogen to freeze thaw cycles in diverse land-use types. APPLIED SOIL ECOLOGY. 2018; 124:211–217. 10.1016/j.apsoil.2017.11.012

[27] Lukas S, Abbas SJ, Karlovsky P, Potthoff M, Joergensen RG. Substrate use and survival of fungal plant pathogens on maize residues at winter temperatures around freezing point. Soil Biology and Biochemistry. 2014; 77:141–149. 10.1016/j.soilbio.2014.06.023

[28] Vu HM, Duman JG. Upper lethal temperatures in three cold-tolerant insects are higher in winter than in summer. The Journal of Experimental Biology. 2017; 220:2726–2732. 10.1242/jeb.161331 28768748

[29] Maqbool MA, Aslam M, Ali H. Breeding for improved drought tolerance in Chickpea (Cicer arietinum L.). Plant Breeding. 2017; 136:300–318. 10.1111/pbr.12477

